# Differences in virulence of pneumolysin and autolysin mutants constructed by insertion duplication mutagenesis and in-frame deletion in *Streptococcus pneumoniae*

**DOI:** 10.1186/1472-6750-14-16

**Published:** 2014-02-21

**Authors:** Esther Yip-Mei Liu, Feng-Yee Chang, Jen-Chang Chang, Chang-Phone Fung

**Affiliations:** 1 Institute of Clinical Medicine, School of Medicine, National Yang-Ming University, Taipei 11217, Taiwan; 2 Division of Infectious Diseases, Department of Internal Medicine, Tri-Service General Hospital, National Defense Medical Center, Taipei 11490, Taiwan; 3 National Institute of Infectious Diseases and Vaccinology, National Health Research Institutes, Zhunan, Miaoli County 35053, Taiwan; 4 Division of Infectious Diseases, Department of Medicine, Taipei Veterans General Hospital and National Yang-Ming University, Taipei 11217, Taiwan

**Keywords:** Insertion duplication mutagenesis, In-frame deletion, Pneumolysin, Autolysin, Target gene restoration

## Abstract

**Background:**

Insertion duplication mutagenesis (IDM) and in-frame deletion (IFD) are common techniques for studying gene function, and have been applied to pneumolysin (*ply*), a virulence gene in *Streptococcus pneumoniae* (D39). Discrepancies in virulence between the two techniques were observed in both the previous and present studies. This phenomenon was also observed during mutation analysis of autolysin (*lytA*).

**Results:**

Our data showed that target gene restoration (TGR) occurred in IDM mutants, even in the presence of antibiotics, while the IFD mutants were stable. In PCR result, TGR occurred later in IDM-*ply* and -*lytA* mutants cultured in non-supplemented medium (4–5 h) compared with those grown in medium supplemented with erythromycin (erm)/chloramphenicol (cat) (3–4 h), but plateaued faster. Real-time PCR for detecting TGR had been performed. When compared with 8-h culture, TGR detection increased from Day 1 and Day 2 of IDM mutant’s culture. erm-sensitive clones from IDM mutant were found. Southern blot hybridization and Western blotting also confirmed the phenomenon of TGR. The median survival of mice following intraperitoneal (IP) injection with a 3-h culture of IDM-mutants was significantly longer than that with an 8-h culture, irrespective of antibiotic usage*.* The median survival time of mice following IP injection of a 3-h culture versus an 8-h culture of IDM-*ply* in the absence of antibiotics was 10 days versus 2 days (*p* = 0.031), respectively, while in the presence of erm, the median survival was 5 days versus 2.5 days (*p* = 0.037), respectively. For an IDM-*lytA* mutant, the corresponding values were 8.5 days versus 2 days (*p* = 0.019), respectively, for non-supplemented medium, and 2.5 versus 2 days (*p* = 0.021), respectively, in the presence of cat. A comparable survival rate was observed between WT D39 and an 8-h IDM culture.

**Conclusion:**

TGR in IDM mutants should be monitored to avoid inconsistent results, and misinterpretation of data due to TGR could lead to important biological meaning being overlooked. Therefore, based on these results, IFD is preferable to IDM for disruption of target genes.

## Background

In the past two decades, advances in gene manipulation technologies have been applied widely in different aspects of molecular research, and have been especially useful in the functional analysis of genes. The analysis of mutant organisms generated by molecular modification is important in determining the functions of the wild-type genes. Homologous recombination in bacterial systems is the main tool for investigation of gene function, and enables the generation of targeted mutants of almost any gene. These techniques have increased our ability to investigate the temporal control of gene knockdown, analyze mutations, and express proteins through targeted gene “knock in” into another gene. Other than transposon mutagenesis and site–specific mutations, two other methods are widely used for functional analysis of bacterial genes: insertion duplication mutagenesis (IDM) and in-frame deletion (IFD) [1-5]. Comparison of a parental bacterial strain and its isogenic knockdown (KD) or knockout (KO) mutant, generated using these techniques, may reveal its specific function.

IDM has been widely used in the study of virulence genes such as pneumolysin (*ply*) and autolysin (*lytA*) in *Streptococcus pneumoniae*[6-11]. In this method, a partial target gene is amplified and ligated into a vector with a resistance marker. The constructed plasmid containing the chimeric DNA sequence then undergoes homologous recombination with the target chromosomal region following transformation. Integration and linearization, followed by insertion of the complete plasmid into the target gene, occurs in a single crossover between the two homologous gene sequences. As a result, the function of the target gene is disrupted [10,12].

The other approach is IFD, which is a replacement homologous recombination [8,13]. Unlike IDM, in which the insertion construct is integrated into the homologous site of the target gene, IFD is preceded by a double cross-over event, leading to the complete replacement of the target gene. As a result, none of the sequence is duplicated in the recombinant, and the wild-type gene cannot be regenerated.

Although both methods, which have been widely used in virulence studies in *S. pneumoniae*, can achieve the goal of gene disruption, no studies have compared whether the experimental data produced by IDM are akin to that produced by IFD. In this study, the stability of IDM and IFD was evaluated. We also examined whether target gene restoration (TGR) could occur in either type of mutant.

## Results

### Confirmation of *S. pneumoniae* IDM-*ply* and -*lytA* mutants

**Figure 1:**
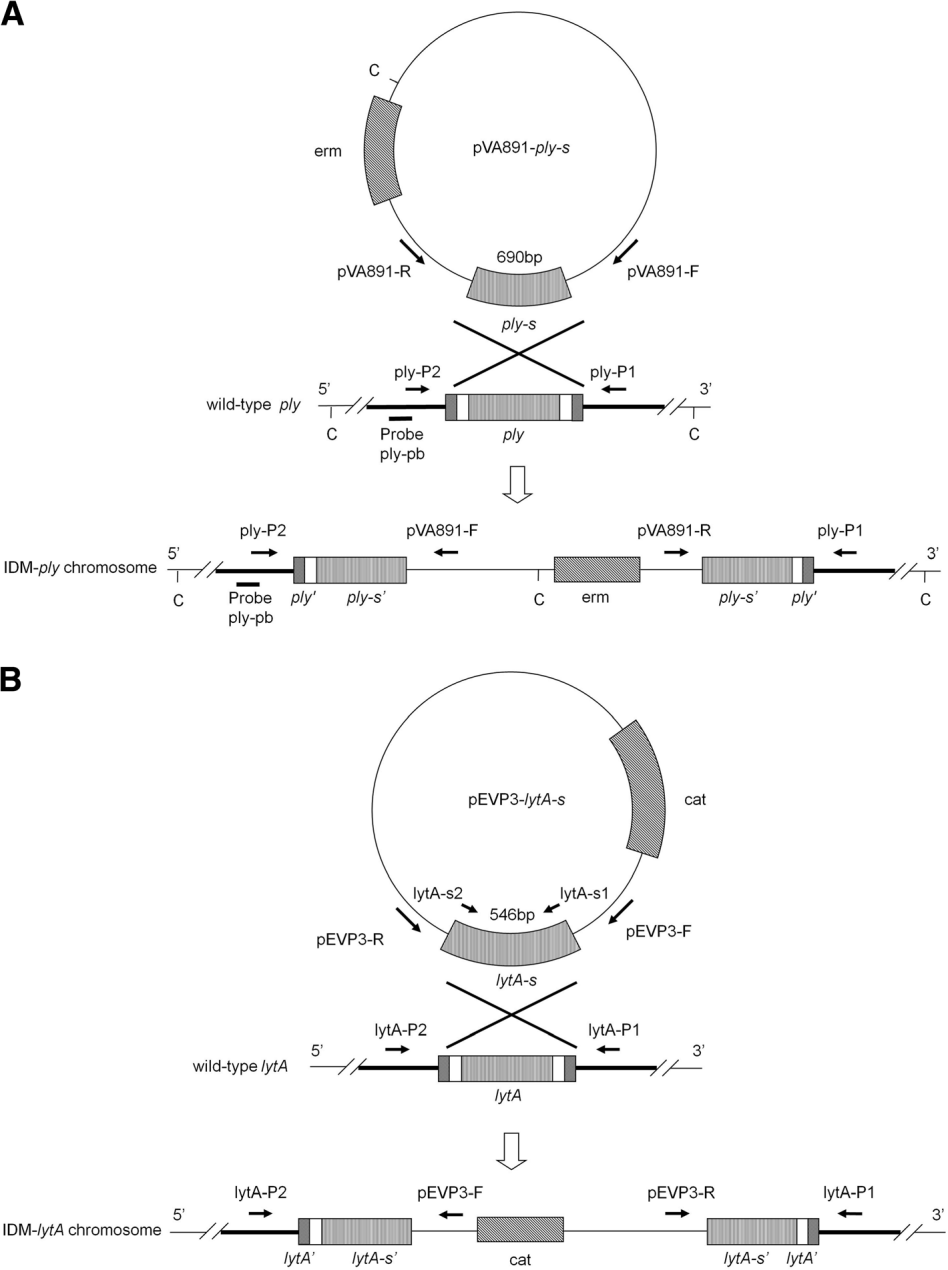
**Construction of the *ply *and *lytA *knockdown mutants by IDM.** Partial sequences of pneumolysin (*ply*-s) **(A)** and autolysin (*lytA*-s) **(B)** genes were cloned into plasmids pVA891 **(A)** and pEVP3 **(B)**, containing an erythromycin (erm)- and a chloramphenicol (cat)-resistance marker, respectively. Cloned plasmids were then used to generate homologous recombinants from their WT *ply* or *lytA* genes, respectively. Two primer sets, ply-P1/pVA891-R and ply-P2/pVA891-F or lytA-P1/pEVP3-R and lytA-P2/pEVP3-F, were used to verify the insertion of pVA891 and pEVP3 in IDM-*ply***(A)** and -*lytA***(B)**, respectively. C, *Cla*I.

**Figure 2:**
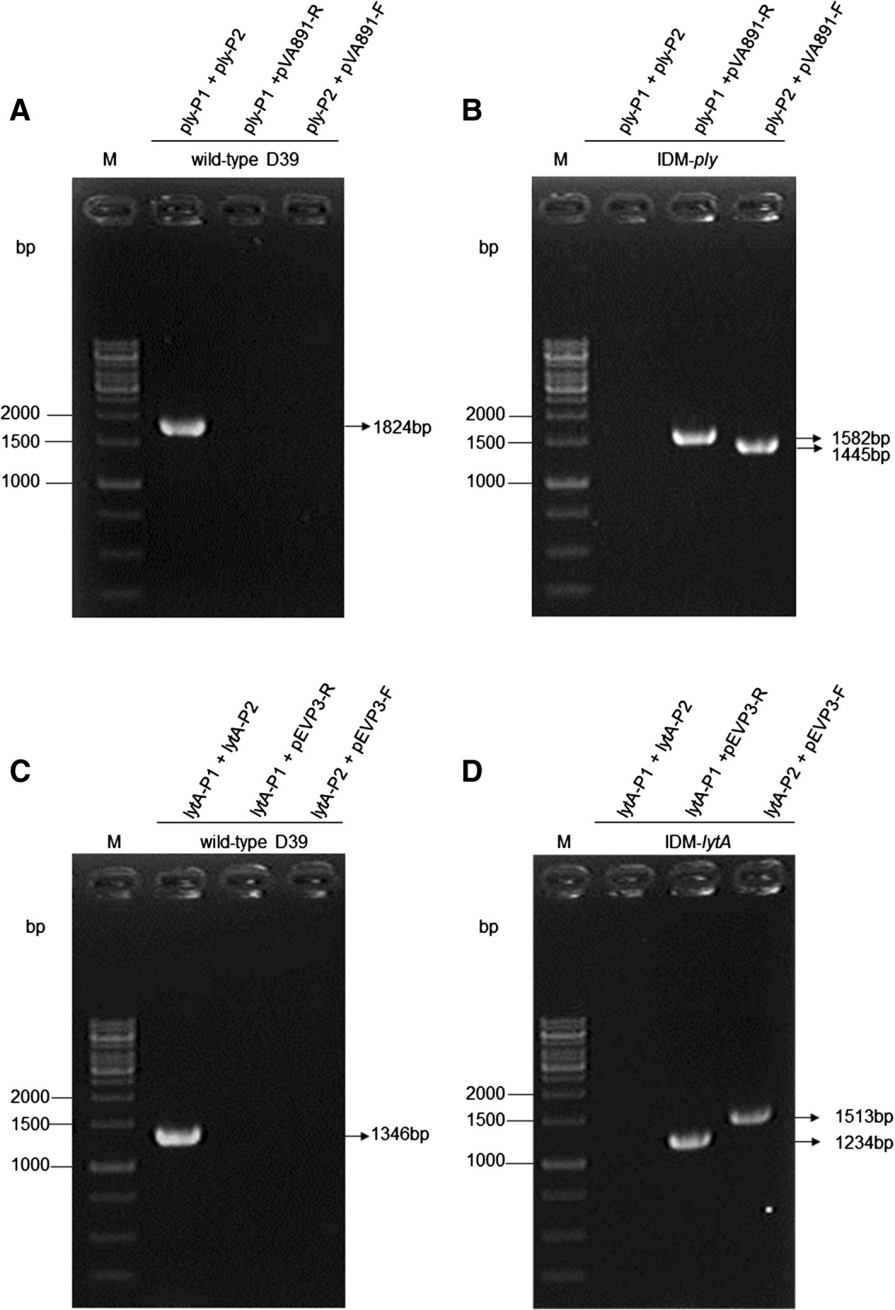
**Verification of D39 and the IDM-*ply *and -*lytA*mutants. (A)** A product could only be amplified from D39 using primer set ply-P1/ply-P2. **(B)** The IDM-*ply* mutant was confirmed by the lack of an amplification product using the ply-P1 and ply-P2 primer set, and was shown to contain an inserted pVA891 and truncated *ply* using primers pairs ply-P2/pVA891-F and ply-P1/pVA891-R, respectively. **(C)** A product was only amplified from D39 using primer pair lytA-P1/lytA-P2. **(D)** The IDM-*lytA* mutant was verified by no amplification product using primers lytA-P1 and lytA-P2 and shown to have an inserted pEVP3 and truncated *lytA* using primer sets lytA-P2/pEVP3-F and lytA-P1/pEVP3-R, respectively. The locations of primers and predicted molecular weight of the PCR products for D39, IDM-*ply*, and IDM-*lytA* are given in Figure 1 and Table 1. M, molecular DNA marker.

Three sets of PCR primers were used to confirm the correct formation of IDM-*ply* mutants (Table 1 and Figure 1A). Positive DNA fragments were observed for IDM-*ply* using the ply-P2/pVA891-F and ply-P1/pVA891-R primer set. As expected, no amplification was observed with the ply-P1/ply-P2 primer set (Figure 2B). Bands were amplified from wild-type (WT) strain D39 using the ply-P1/ply-P2 primer set, but not with the ply-P2/pVA891-F and ply-P1/pVA891-R primer set (Figure 2A).

**Table 1:** **Primers used to assess and confirm the mutants for IDM- *ply *, IDM- *lytA *, IFD- *ply *, and IFD-*lytA***

	**Sequence (5′–3′)**	**Accession number**	**PCR product size (bp)**		
			**D39**	**IDM**	**IFD**
Pneumolysin					
ply-P1	CCTGTATATCAAGGAATTGGCTACC	CP000410.1			
ply-P2	GATAGAAGAGCCGGATCTAGCTCG	CP000410.1	1824	ND	670
ply-P1	CCTGTATATCAAGGAATTGGCTACC	CP000410.1			
pVA891-R^a^	CGCTCATCGTCATCCTCGGC	AB057644.1		1582	
ply-P2	GATAGAAGAGCCGGATCTAGCTCG	CP000410.1			
pVA891-F^a,^	CCAGTAGTAGGTTGAGGCCGTTG	AB057644.1		1445	
ply-1a	CCTTTGGCTTTATCAATCGCTTTATCG	CP000410.1			
ply-1b	actcttcacttacatgcatgcCGTACGGTTTATGAAAAAACC	CP000410.1			1227
ply-2a	gcatgcatgtaagtgaagagtGCTCCGCTTCTTTCTTTCGAT	CP000410.1			
ply-2b	TTACCTGTCGCCCTTGCTC	CP000410.1			1185
ply-F91	CGTTTCATCAAAGAGGGTAA	CP000410.1			
ply-R1070	GCTGTAACCTTAGTCTCAAC	CP000410.1	980		
ply-pbF	TAGCGATTTGGCTGTGAA	CP000410.1			
ply-pbR	GCTGATTACCCTCTTTGA	CP000410.1	483		
Autolysin					
lytA-P1	CAGTACTTAAAGCTATCCGACTCGGTTTAC	CP000410.1			
lytA-P2	GTCTGGGGTGTTATTGTAGATAGAATG	CP000410.1	1346	ND	616
lytA-s1	CATGGCGCCTTCTTTAGCGTCTA	CP000410.1			
lytA-s2	GAGTTCATGACGGACTACCGCCT	CP000410.1		546	
lytA-P1	CAGTACTTAAAGCTATCCGACTCGGTTTAC	CP000410.1			
pEVP3-R^b^	GCCAGTTTGAGGGGACGACG			1234	
lytA-P2	GTCTGGGGTGTTATTGTAGATAGAATG	CP000410.1			
pEVP3-F ^b^	TAACCTAACTCTCCGTCGC			1513	
lytA-1a	GTTCCTCATACTTCCCTTG	CP000410.1			
lytA-1b	cccatccactaatgcggccgcATGCCTTTATCCAGTCAGCGG	CP000410.1			1331
lytA-2a	gcggccgcattagtggatgggATTCCCAGTTGAGTGTGCGTG	CP000410.1			
lytA-2b	TGCCAGAACTCTTGCCACAG	CP000410.1			1199
Glucose kinase					
gki-up	GGCATTGGAATGGGATCACC	CP000410.1			
gki-dn	TCTCCCGCAGCTGACAC	CP000410.1	626	626	626
gki-F603	TGCCGATGAATACGAAGG	CP000410.1			
gki-R791	CGATTGTTGATGGGTTTAG	CP000410.1	189		

^a^According to pVA838 (GenBank accession number AB057644.1).

^b^pEVP3 was provided by Dr. Don Morrison, UIC Biological Sciences, 900 South Ashland Ave., Chicago, IL 60607, USA.

ND Not detectable.

Nucleotides shown in lowercase letters denote extra sequences that were added for ligation reactions.

**Figure 3:**
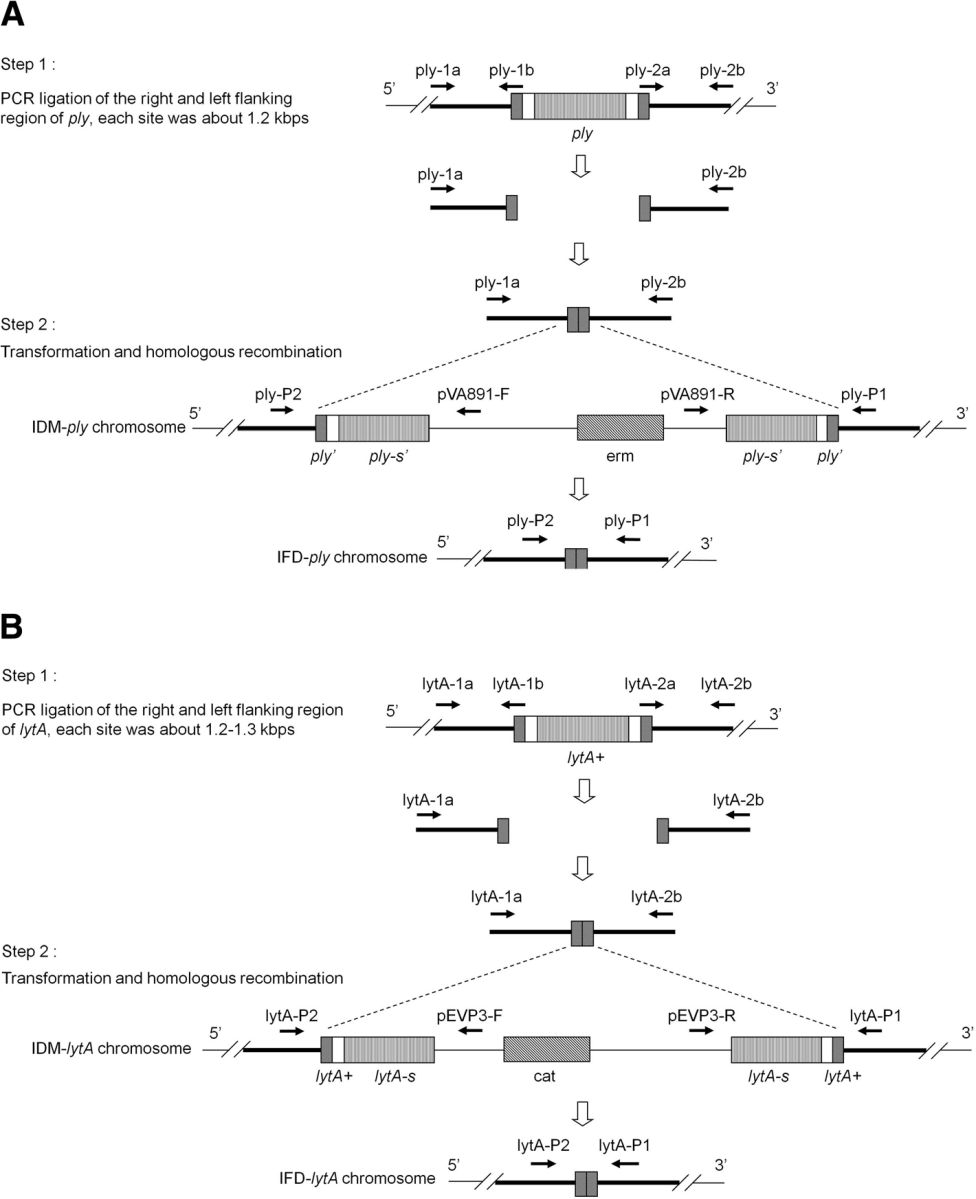
**Construction of the *ply *and *lytA *knockout mutants by IFD.** In step 1, linear DNA fragments containing flanking regions of the *ply***(A)** and l*ytA***(B)** genes, generated by PCR, were transformed into IDM-*ply***(A)** and -*lytA***(B)**, respectively, for a second round of homologous recombination. The second recombination event could result in either restoration of the original gene, or mutants of IFD-*ply***(A)** and -*lytA***(B)** with a double-crossover. IFD-*ply***(A)** and -*lytA***(B)** mutants were selected by decreasing concentration of erythromycin (erm) for *ply* or chloramphenicol (cat) for *lytA*, and increasing concentrations of ampicillin. Primer sets ply-p1/ply-P2 or lytA-P1/lytA-P2 were used to verify the IFD mutants.

Locations of primers are shown in Figure 1 for IDM and Figure 3 for IFD.

Incorporation of pEVP3-chloramphenicol (cat), carrying a partial *lytA* sequence, into target chromosomal *lytA* by homologous recombination, generating a IDM-*lytA* mutant, was confirmed using three PCR primer sets (Table 1 and Figure 1B). DNA fragments were obtained from IDM-*lytA* using the lytA-P2/pEVP3-F and lytA-P1/pEVP3-R primer set. No DNA amplification was observed from IDM-*lytA* DNA using the lytA-P1/lytA-P2 primer set (Figure 2D). Positive DNA fragments were only obtained from WT D39 DNA using the lytA-P1/lytA-P2 primers, while no amplification was observed using the lytA-P1/pEVP3-R and lytA-P2/pEVP3-F primer sets (Figure 2C).

### Confirmation of *S. pneumoniae* IFD-*ply* and -*lytA* mutants

**Figure 4:**
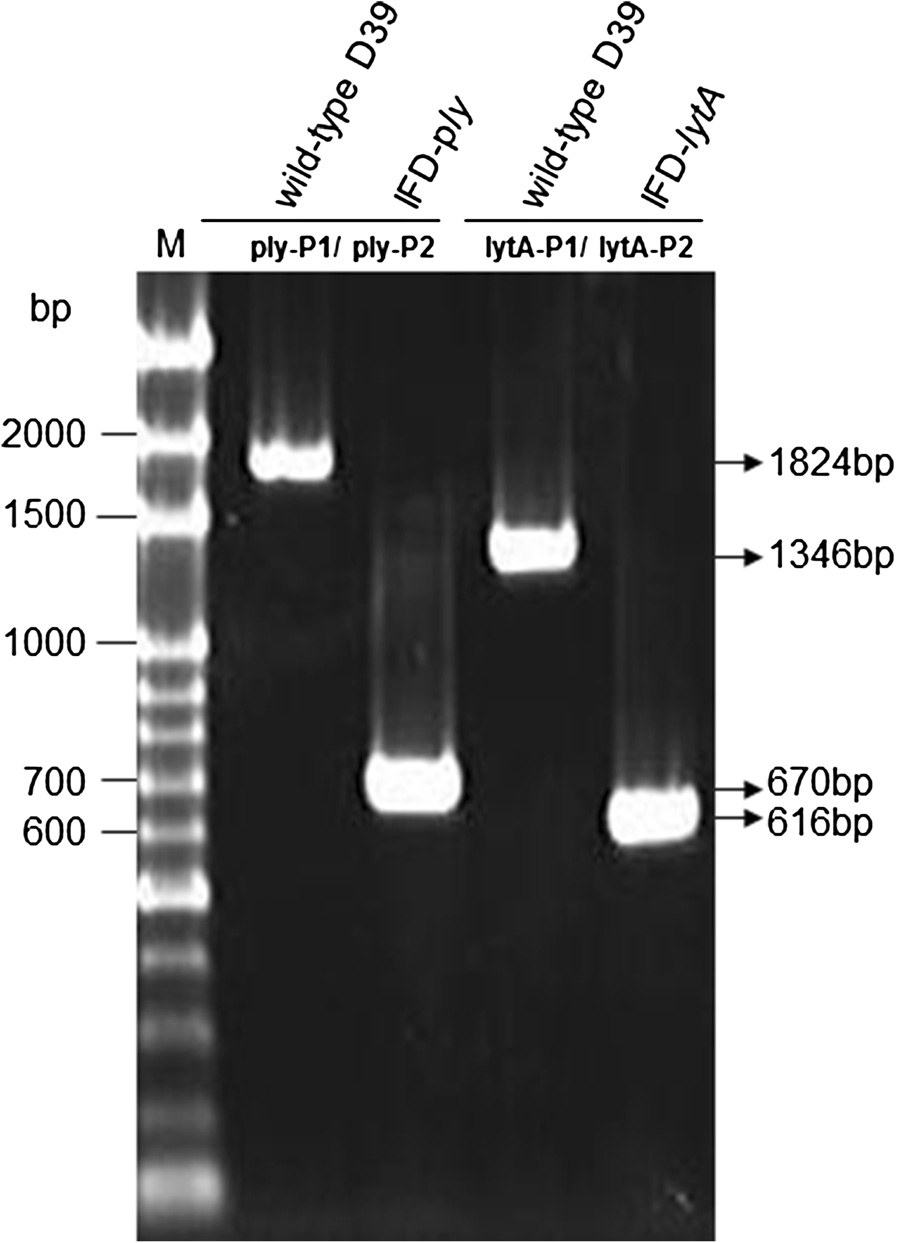
**Verification of D39 and the IFD-*ply *and -*lytA *mutants.** PCR using different primer sets specific to *ply* and *lytA* were used to confirm parental D39 and the IFD-*ply* and -*lytA* mutants. The locations of primers and predicted molecular weights of the PCR products for D39, IFD-*ply*, and IFD-*lytA* are given in Figure 3 and Table 1. M, molecular DNA marker.

By transformation of linear DNA fragments comprising the 5′ and 3′ flanking regions of *ply* or *lytA* (Figure 3A and B), respectively, into IDM-*ply* and -*lytA*, IFD-*ply* and -*lytA* mutants were obtained from a second round of homologous recombination. Both IFD mutants were confirmed by PCR using primer sets ply-P1/ply-P2 for pneumolysin and lytA-P1/lytA-P2 for autolysin (Figure 3A and B). WT strain D39 was used as a control. Predicted PCR products are shown in Table 1. A 670-bp and a 616-bp fragment were obtained for the IFD-*ply* and IFD-*lytA* mutants, respectively, using the same primer pairs as above, while positive amplification of D39 produced a 1824-bp fragment for *ply* and a 1346-bp fragment for *lytA* (Figure 4).

### Detection of TGR in IDM and IFD mutants by PCR

**Figure 5:**
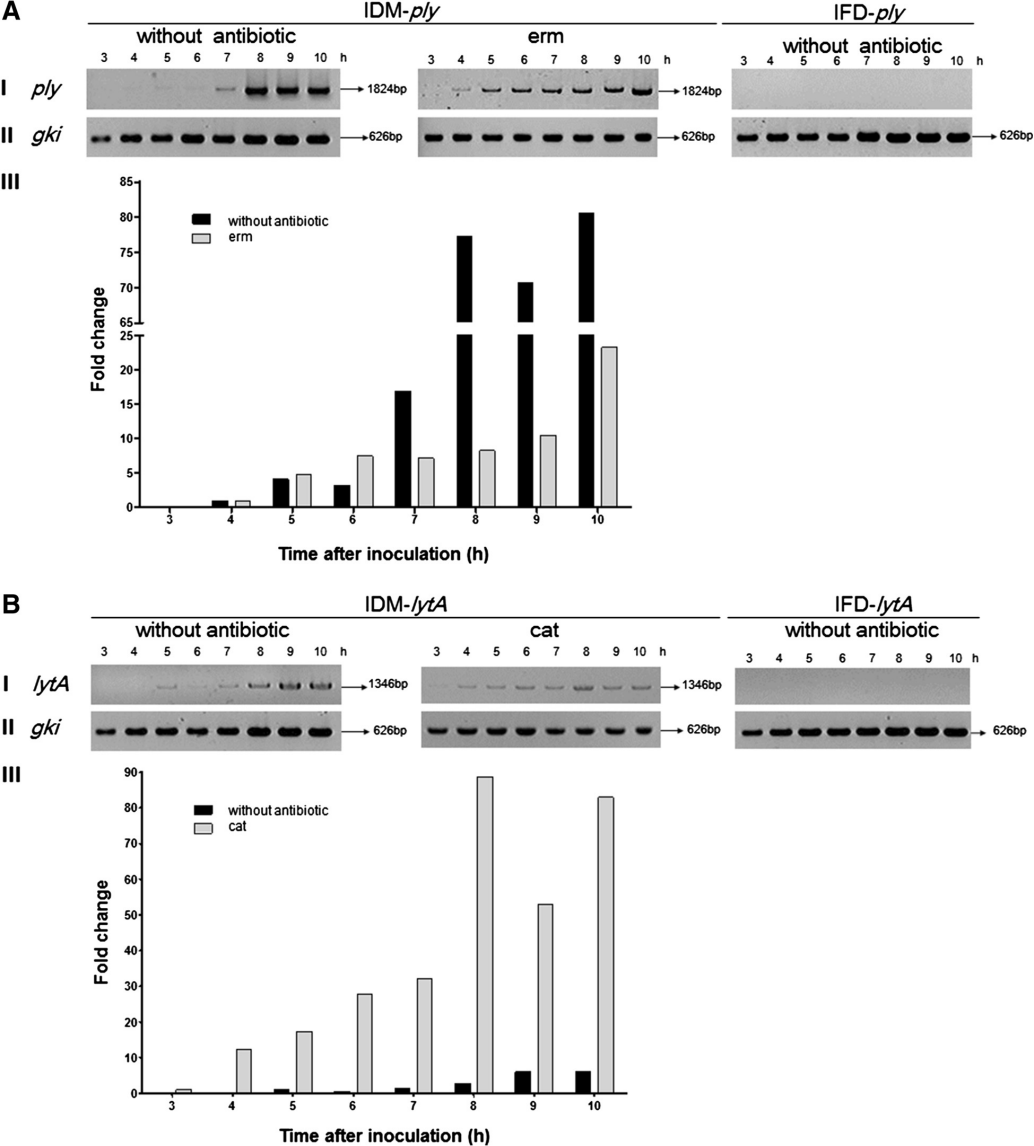
**Target gene restoration (TGR) of wild-type *ply *(A) and *lytA *(B) was only observed in IDM mutants.** (I) Full-length target genes were amplified by PCR of samples taken at 3–10 h post-inoculation. Restoration of *ply***(A, I)** and *lytA***(B, I)** was confirmed by PCR using the primer sets ply-P1/ply-P2 and *lytA*-P1/*lytA*-P2, respectively, in 3–10-h cultures of IDM-*ply*/-*lytA* with and without antibiotic selection. No target gene restoration was observed in IFD-*ply*/-*lytA* mutants. (II) Housekeeping gene *gki*, used as a loading control to evaluate the level of gene expression, was amplified by primer pair gki-up/gki-dn. (III) Fold change refers to ratio of the target gene restoration (*ply* or *lytA*) to *gki* in 3–10-h cultures over the aforementioned ratio found at the earliest time of target gene detection.

TGR was identified in both the IDM-*ply* and -*lytA* mutants, while no TGR was found in either the IFD-*ply* or -*lytA* mutants (Figure 5AI and BI). WT *ply*, formed by TGR in IDM-*ply,* was observed 4–5 h post-inoculation of mutants into plain brain-heart infusion (BHI) broth under antibiotic selection (Figure 5AI). This restoration of mutants back to the WT genotype increased to a plateau at about 8–10 h of cultivation, with or without antibiotic selection (Figure 5AI). The highest percentage increase in *ply* detection, relative to the earliest detection of TGR (4 h), in plain BHI and BHI with erythromycin (erm) was 80.57% and 23.27%, respectively, indicating a relatively slow rate of TGR under antibiotic selective pressure (Figure 5AIII). TGR was also observed in IDM-*lytA* mutants (Figure 5BI). In BHI medium, TGR was first detected at around 5 h post-inoculation and corresponded to a 6-fold change in detection, while in BHI supplemented with cat, TGR was detected at 3 h post-inoculation and corresponded to a 90-fold change in detection (Figure 5BIII).

### Validation of TGR from the results of previous PCR assay of IDM mutants by using real-time PCR assay, southern blot hybridization and western bloting

**Figure 6:**
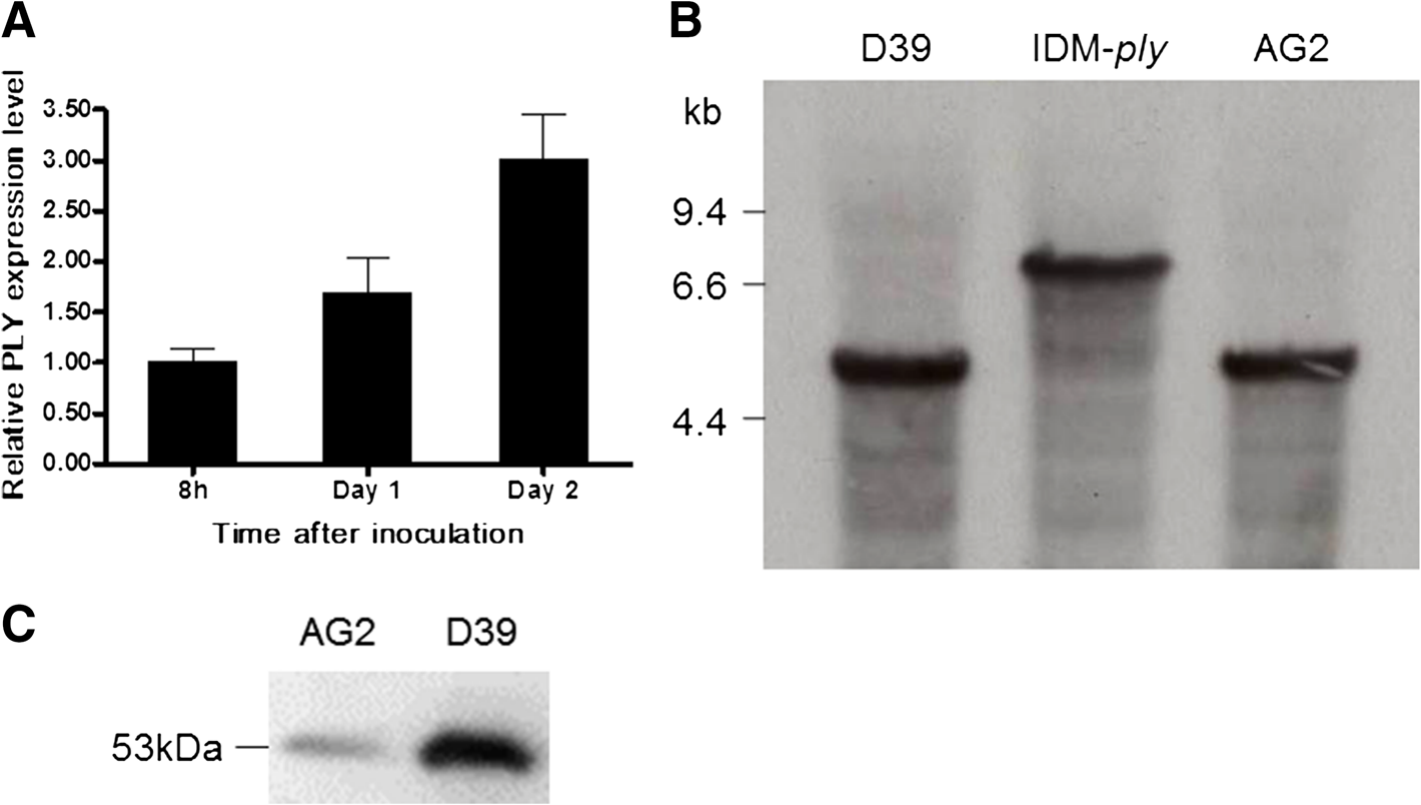
**Validation of TGR of WT-*ply *from IDM-*ply *mutant. (A)** Expression of WT-PLY in the 8 h, Day 1 and Day 2 IDM-*ply* culture by real-time PCR **(B)***Cla*I- digested D39, IDM-*ply* and AG2, which was sensitive to erm and was obtained from Day 1 culture of IDM-*ply* mutant, were probed with a digoxigenin-labeled ply-pb in southern blot hybridization. **(C)** AG2 would produce PLY whose size was the same as PLY of D39 when incubating with a rabbit polyclonal PLY antibody.

Since only PLY antibody is commercial available for the determination of protein expression for TGR strain, validation of TGR from the results of the above PCR assay was used the PLY as a model. In Real time PCR assays, full length PLY expression level of Day 1 and Day 2 culture of IDM-*ply* was compared 8-h culture of IDM-*ply*. A 1.66 and 3.00 folds were respectively observed in Day 1 and Day 2 culture of IDM-*ply* indicating TGR of PLY occurred as time dependent manner (Figure 6A). In Southern blotting, *erm* susceptible isolates were selected from Day 1 culture of IDM-*ply* and hybridization with *pl*y specific probe confirmed that TGR wild type strains was occurred (Figure 6B). For determination of full length protein expression of PLY, Western blotting was performed and confirmed that the PLY expression in TGR was as same as wild type D39 (Figure 6C).

### Effect of TGR on virulence of mutants in a mouse model of infection

**Figure 7:**
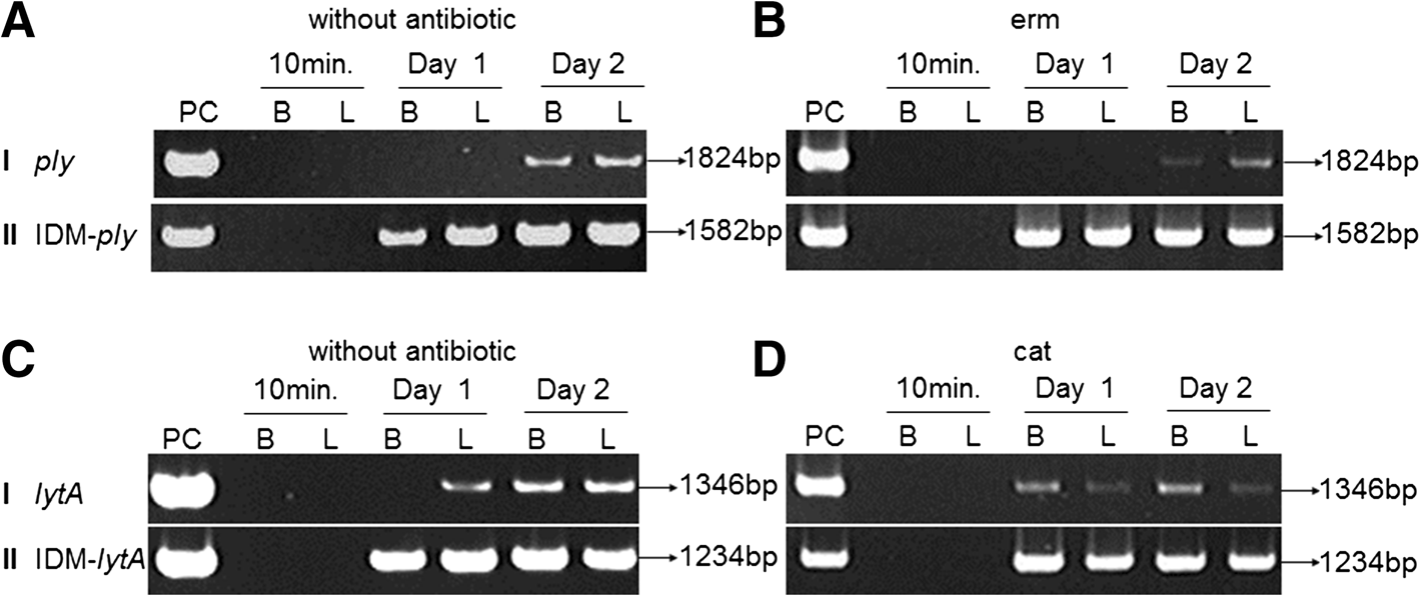
**Detection of TGR of WT*ply*** **and *lytA*** **gene from blood and liver of BALB/c mice with 3h-IDM culture of IDM mutants. (A-D)** Cultural media used for IDM mutants growth. IDM-*ply* mutant was cultivated in plain BHI broth **(A)** and BHI supplemented with erm **(B)** whilst IDM-*lytA* mutant was grown in plain BHI broth **(C)** and BHI supplemented with cat **(D)**. (I) Detection of the restored WT *ply*/*lytA* gene from blood and liver samples following IP injection of 3-h culture of IDM-*ply*/-*lytA* mutants at 10min, 1 day and 2 days. WT *ply* gene was amplified from samples by using the ply-P1/ply-P2 primer set for IDM-*ply* mutant grown from plain BHI (A,I) and BHI with erm (B,I). Full length of *lytA* gene was detected by the lytA-P1/lytA-P2 primer set for IDM-*lytA* mutant grown from BHI (C,I) and BHI with cat (D,I). (II) PCR analysis of IDM-*ply/lytA* gene from the same samples used in (I). Detection the genes of IDM-*ply* from IDM*-ply* mutant grown from BHI (A,II) and BHI with erm (B,II), and IDM–*lytA* gene from IDM-*lytA* mutant grown from BHI (C,II) and BHI with cat (D,II) was performed by ply-P1/pVA891-R and lytA-P1/pEVP3-R respectively. The lowest inocula of the 3-h IDM-*ply* cultures in BHI alone and BHI supplemented with erm for positive TGR of *ply* were approximately 10^4^ CFU **(A)** and 10^3^CFU **(B)** respectively whilst that of the IDM-l*ytA* mutant grown in BHI and BHI supplemented with cat for positive TGR of *lytA* were 10^2^ CFU **(C)** and 10^3^ CFU **(D)** respectively. B, blood; L, liver; erm, erythromycin; cat, chloramphenicol.

For the *in-vivo* studies, mice were randomly selected and sacrified for detection of *ply* and *lytA* by PCR*.* No mice showed symptoms of illness at 10 min post-injection (IP), and neither the WT D39 nor the IDM mutant strain was found in the heparinized blood or liver at this time point (Figure 7A–D). However, full-length *ply* was consistently detected in the blood and liver of animals injected with IDM mutants grown with or without antibiotics at day 2 (Figure 7A and B), and full length *lytA* was also detected as early as day 1 (Figure 7C and D). IDM-*ply* and -*lytA* were consistently detected in all samples on day 1, indicating the coexistence of IDM mutants and TGR WT D39 during infection (Figure 7A–D).

**Figure 8:**
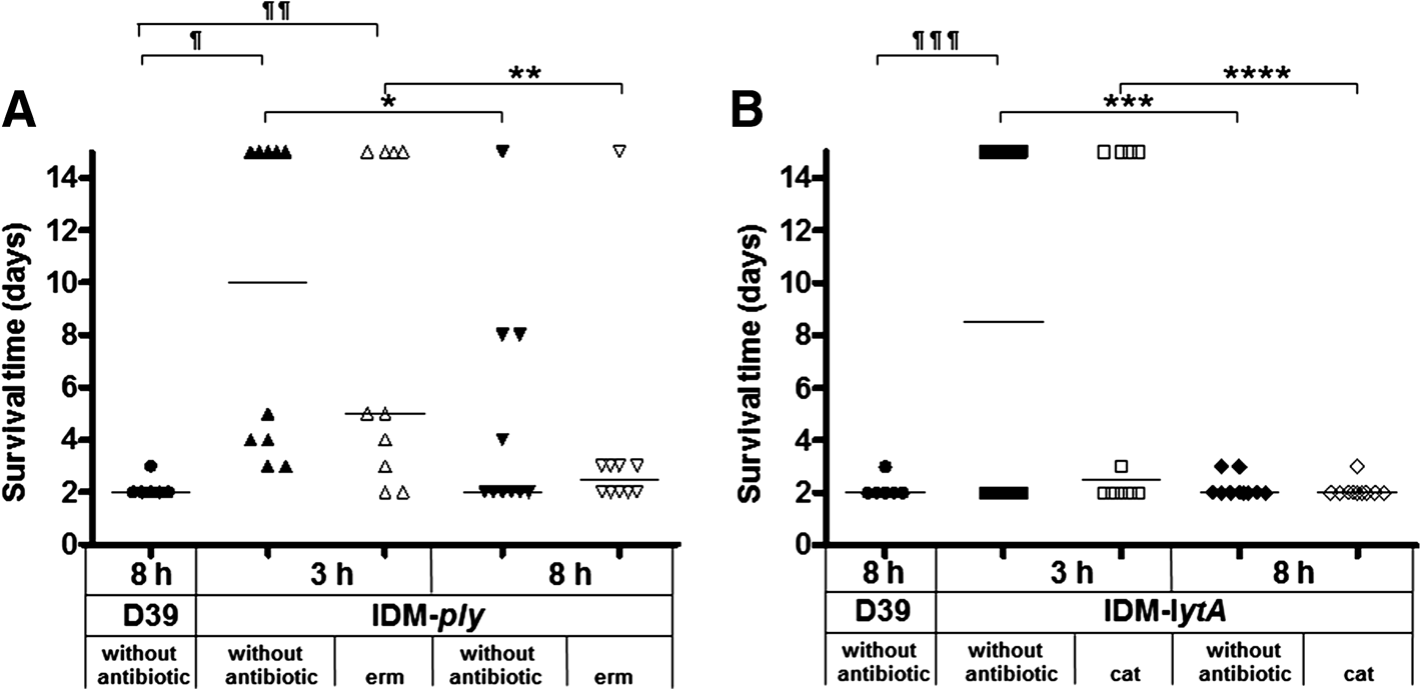
**Survival of BALB/c mice injected intraperitoneally with WT D39 and a 3-h and an 8-h culture of IDM-*ply *(A) and -*lytA *(B).** All of the inocula were approximately 10^2^ CFU. The median survival time is indicated by the dashed line. Significant differences in the median survival time of mice injected with a 3-h IDM-*ply***(A)** or -*lytA***(B)** culture versus those for mice injected with an 8-h culture of the IDM- mutants are indicated by an asterisk (*) (*p = 0.031 (without antibiotic) versus **p = 0.037 (erm usage) in IDM-*ply*; ***p = 0.019 (without antibiotic) and ****p = 0.021(cat usage) in IDM-*lytA*). Significant differences between mice injected with 8-h WT D39 and those injected with 3-h IDM clones are indicated by a pilcrow (^¶^). (^¶^*p* < 0.001(without antibiotic) and ^¶¶^*p* = 0.006 (erm usage) when compared to the 3-h IDM-*ply*; ^¶¶¶^*p* = 0.045 (without antibiotic) when compared to the 3 h IDM-*lytA*).

Approximately 10^2^ colony forming units (CFU) of IDM-*ply* mutant was then used in a lethality test*.* The median survival time of mice injected with a 3-h IDM-*ply* culture was significantly longer than that of animals injected with an 8-h IDM-*ply* culture: 10 versus 2 days, *p* = 0.031, without antibiotic selection; 5 versus 2.5 days, *p* = 0.037, with erm selection (Figure 8A). Following IP injection of a 3-h IDM-*ply* culture grown with and without antibiotics, five and four animals, respectively, out of 10 survived after 14 days of observation. All except one mouse (90%) died within 14 days when an 8-h IDM-*ply* culture was used. The survival time following injection with a 3-h IDM-*ply* culture was significantly longer (*p* < 0.001 without antibiotic and *p* = 0.006 with erm) than that with an 8-h culture of WT D39, indicating decreased virulence of the 3-h IDM-*ply* mutant. A comparable survival rate was observed between WT D39 and an 8-h IDM-*ply* culture (Figure 8A), indicating a high level of TGR in the 8-h IDM-*ply* culture.

Similarly, the median survival time of mice subjected to IP injection with 10^2^ CFU of a 3-h culture of IDM-*lytA* was significantly longer than that following injection with the same amount of an 8-h IDM-*lytA* culture (8.5 vs. 2 days, *p* = 0.019 with no antibiotic; 2.5 vs. 2 days, *p* = 0.021 with cat) (Figure 8B). The survival rates for the two groups were 50% and 40%, respectively, at 14 days post-inoculation. None of the mice survived longer than 3 days post-inoculation with an 8-h culture of IDM-*lytA*. Like the IDM-*ply* mutant, the 3-h IDM-*lytA* culture had attenuated virulence in mice, demonstrated by a decreased mortality rate (*p* = 0.045 for non-supplemented BHI), when compared to the 8-h D39 culture. Overall, the 3-h IDM mutants were less virulent than the 8-h IDM mutants. The survival of mice injected with 8-h IDM-*ply* or -*lytA* culture was almost identical to that of mice injected with WT D39.

## Discussion

A search of the literature shows that IDM is still a commonly used method for gene KD [14], and has been used to study pneumolysin and autolysin in *S. pneumoniae*[15-17]. In the present study, TGR in IDM mutants caused by intra-chromosomal recombination of a mutated target gene, was confirmed and significantly affected lethality in mice. Selective antibiotic pressure minimized the extent of the TGR but could not completely eliminate the rearrangements. Higher concentrations of antibiotics could prohibit reversion, but the growth rate of the bacteria was significantly affected. Re-circularization of inserted sequence within the IDM mutants circumvents the antibiotic selection. This type of mutant maintains its antibiotic resistance but loses the KD function of the target gene. Following injection of these mutants into mice, antibiotic selective pressure is relaxed, leading to an increased chance of TGR. This event was observed in our animal study, in which TGR-D39-type *ply* and *lytA* gene sequences were detected at days 2 and 1 post-injection, respectively (Figure 7). This effect generated the discrepancy in pathogenicity and lethality in mice, and affects the interpretation of results in our study (Figure 8).

A previous study also showed decreased survival of mice injected IP with IDM-*ply* compared with those injected with IFD-*ply*[8]. The median survival times of mice injected with IDM-*ply* (“PLN-A”) and IFD-*ply* (“ΔPly”) were 2.8 and 13.8 days, respectively, with a *p-*value of 0.04 (log-rank survival test), suggesting that the IDM-*ply* mutants were significantly different from the IFD-*ply* mutants, and were more virulent. Polar effects of the pVA891-mediated IDM event, along with toxicity of truncated pneumolysin polypeptides or fusion proteins resulting from insertion of the plasmid sequences into the *ply* gene cannot be excluded [8]. Our data proved that the relatively greater virulence of the IDM mutants in the mouse model may also due to TGR. The restoration of the WT gene is not specific to *ply* or plasmid pVA891, as it also occurred with the virulence gene *lytA* and plasmid pEVP3. We have previously observed this phenomenon in Gram-negative bacteria when we applied IDM to KD *ompK36,* an outer membrane porin gene of *Klebsiella pneumoniae*[18]. Nonetheless, this gene restoration effect is not common to all genes. No target gene restoration was observed in IDM-*wzy*_KPK1_, a capsule synthesis polymerase gene in *K. pneumoniae*[19]. Our PCR analysis of TGR of *lytA* IDM mutants showed that TGR could be detected at as early as 3-h post-inoculation, supporting the discrepancy in results when an 8-h culture was selected for injection. Our study of lethality showed that the median survival time (2 days) of mice when using the 8-h IDM mutants was comparable to direct IP injection of the original D39 strain. We postulated that the IDM mutants could revert to their parental characteristics, resulting in WT levels of virulence (Figure 8).

Based on our previous experience with IDM-*ompK36*, TGR most likely occurred at a certain time during culture in broth medium, and the effect would not be detectable at culture times of less than 2 h [18]. However, the consequence of not using serial passages of such mutants has given rise to huge inconsistencies in the data. Because the growth rate of *S. pneumoniae* is significantly slower than many other Gram-positive bacteria in culture media, the problems associated with the unstable mutation become substantial when attempting to generate a considerable concentration of the mutant. Although this difficulty can be overcome by PCR confirmation prior to the use of cultured mutants in each experiment, it becomes labor intensive in *in vivo* studies.

In addition to the other known defects of IDM methods, TGR should be confirmed to avoid inconsistent results prior to interpretation and reporting. Misinterpretation of data due to TGR may have contributed to important biological meaning being overlooked. If inconsistencies are observed in IDM-related experimental data, previous data should be retrieved and examined for the possibility of TGR. However, the polar effect, truncated target polypeptides or fusion proteins resulting from insertion of the plasmid sequences of IDM are always the consideration of this method even TGR will not be occurred. The use of IFD to generate mutants in both our previous work [20] and the present study has given consistent data for both *in vitro* and *in vivo* experiments.

## Conclusion

IFD is a superior KD method compared with IDM, as it produces more stable clones, even though the success rate is lower.

## Methods

### Bacterial isolates

Capsular type 2 *S. pneumoniae* strain D39 and a derivative of the D39 pneumolysin knock down mutant (IDM-*ply*), originally described by Berry et al. [7] and constructed using IDM with a defined truncation in the pneumolysin gene (Figure 1A), were kindly provided by Dr. David Briles, University of Alabama, Birmingham, AL, USA [21].

### Construction of the autolysin KD mutant (IDM-*lytA*) by IDM

The *lytA* mutant was constructed by IDM (Figure 1B). A 546-bp DNA fragment of the autolysin gene was PCR-amplified from D39 using the primer set lytA-s1/lytA-s2 (Table 1). PCR amplification was performed in a DNA thermal cycler (Perkin-Elmer Biosystems, Foster City, CA, USA) in a 50-μl mixture containing Phusion HotStar High-Fidelity DNA polymerase, 200 μM deoxynucleotide triphosphates (GeneTeks BioScience, Taipei, Taiwan), and 0.6 μM of each oligonucleotide primer in 1× Phusion HF buffer. Template DNA (20 ng) was added to 48 μl of the master mix. The amplification profile included an initial denaturation step at 98°C for 30 s, followed by 35 cycles of 98°C for 10 s, 58°C for 10 s, and 72°C for 1 min, and a final extension of 72°C for 10 min. The suicide vector pEVP3 [22] was separately digested by *Sma*I (New England Biolabs, Ipswich, MA, USA). The pEVP3/*Sma*I digest was treated with calf intestinal alkaline phosphatase (New England Biolabs), and then ligated with the blunt-ended amplicons using T4 DNA ligase (New England Biolabs) according to the manufacturer’s instructions. Transformation and homologous recombination were performed as previously described [23]. WT D39 competent cells were used as the recipient cells. Mutants with plasmid integrated into *lytA* were selected on agar plates containing 3 μg ml^-1^ cat (Figure 1B) [24].

### Verification of the IDM-*ply* and -*lytA S. pneumoniae* mutants

To confirm the successful generation of *ply* and *lytA* knockdown mutants by the IDM technique, PCR amplification of the target sequences was performed. The primers used and predicted sizes of the PCR products of the two IDM mutants are listed in Table 1, and are shown in Figure 1A and B. For IDM-*ply*, three sets of primers were used to verify the parental and the mutant strains: (1) ply-P1/ply-P2, (2) ply-P2/pVA891-F, and (3) ply-P1/pVA891-R. Similarly, three primer sets were used to verify the IDM-*lytA* strain: (1) lytA-P1/lytA-P2, (2) lytA-P2/pEVP3-F, and (3) lytA-P1/pEVP3-R.

### Construction and verification of IFD-*pl*y and -*lytA* mutants

To construct the IFD mutants IFD-*ply* and -*lytA* of D39, overlap extension PCR was used to generate a linear DNA fragment containing the 5′ and 3′ flanking regions of *ply* or *lytA* (Figure 3A and B) [25,26]. Primers used to generate the DNA fragments are shown in Table 1, and the IFD mutants are shown in Figure 3A and B. Following PCR confirmation of the desired linear DNA fragments consisting of the flanking regions of *ply* or *lytA*, homologous recombination was performed using the IDM-*ply* and -*lytA* mutants as recipients. Transformation and homologous recombination for IFD were performed as previously described [23] (Figure 3A and B). Selection of IFD mutants was performed using blood agar plates with decreasing concentrations of erm for *ply* or cat for *lytA*, and increasing concentrations of ampicillin [8].

To confirm the IFD-*ply* and -*lytA* mutants were successfully obtained, PCR amplification of the target sequence was performed. Two pairs of primers, ply-P1/ply-P2 and lyt-P1/lytA-P2, were used to verify IFD-*ply* and -*lytA*, respectively. The predicted sizes of the amplicons are listed in Table 1.

### *In vitro* evaluation of TGR from the IDM or IFD mutant strains

To evaluate TGR, a single colony of each of the IDM mutants, picked from Muller-Hinton agar (MHA) plates supplemented with 5% defibrinated sheep blood and 2.5 μg ml^-1^ erm for the IDM-*ply* mutant or 3 μg ml^-1^ cat for IDM-*lytA*, was simultaneously incubated in 20 ml non-selective BHI medium, to mimic *in vivo* conditions in mice, and in 20 ml BHI with the corresponding antibiotic to compare differences in the rates of TGR. All cultures were incubated at 37°C in 5% CO_2_. Samples were collected hourly from each culture over an 8-h period, beginning at 3 h post-inoculation. Genomic DNA was extracted from these samples using a Gentra Puregene DNA Purification Kit (Qiagen, Hilden, Germany). PCR detection of IDM and IFD mutants was performed immediately after sub-culturing of the mutants into BHI broth to confirm that there was no contamination with the WT D39 in the culture. The primers used to confirm the IDM and IFD mutants are listed in Table 1.

The rate of TGR was measured by semi-quantitative PCR, in which 20 ng of each template DNA was used for each measurement. The glucose kinase gene (*gki*) was used as an internal control and was amplified by primers gki-up and gki-dn (Table 1). Fold change represents the ratio of band intensity of *ply* or *lytA* having been reverted by TGR at different time points (T), divided by that of *gki* at the same time point, against the aforementioned ratio of the genes at the earliest time of TGR detection [(*ply*_T_/*gki*_T_)/ (*ply*_earliest time_/*gki*_earliest time_)]. To ensure no WT D39 contamination, PCR amplification of *ply* and *lytA* was performed concomitantly following sub-culturing of IDM and IFD mutants in BHI broth.

### Validation of TGR from the results of previous PCR assay of IDM mutants by using real-time PCR assay, southern blot hybridization and western blotting

For Real time PCR assay, the 8-h culture, Day 1 and Day 2 culture of IDM-*ply*, cultured as previously mentioned, were collected. RNA was extracted by RNeasy® plus mini kit (Qiagen, Hilden, Germany) and then were converted to cDNA by SuperScipt® III First-strand Synthesis (Life Technologies). qPCR were performed by Step One™ Step One Plus™ 7500 Fast AB Biosystems and Fast SYPR Green Master Mix (Applied Biosystems, USA) and each samples were run in triplicate. Primers ply-F91 and ply-R1070 were used to detect the TGR *ply* whilst endogenous *gki* were detected by gki-F603 and gki-R791 (Table 1).

In Southern blotting, IDM-*ply* growth from different time points were collected. Serial dilution of the cultures were spread to BA to eneumate the viable colonies. Replica-plating with agar with and without erm were performed to select the colony with TGR of WT *ply.*

Southern blotting were performed similar as Ma L et al. [27]. Briefly, 10 μg of genomic DNA from D39 and TGR strain were extracted by Gentra Puregene Yeast/Bact. Kit (Qiagen, Hilden, Germany) and were then digested by *Cla*I (New England Biolabs, Ipswich, MA, USA) according to the manufactures’ instruction. Samples were electrophoresed on an 0.8% agarose, which was then denatured and neutralized. Their DNA fragments were then transferred to a nylon membrane (PerkinElmer). Digoxigenin (DIG)-labeled probe ply-pb, synthesized by PCR amplification of ply-pbF and ply-pbR, were hybridized to the membrane and detected by DIG-luminescent detection kit (Roche, Mannheim Germany) (Table 1). If positive signal will be observed at 5 kb, TGR of full length of *ply* existed. In contrast, band showed at 7 kb indicating an IDM-*ply* mutant.

For the determination of protein expression of *ply*, the expression of WT PLY from IDM-*ply* mutants was checked by blotting of rabbit polyclonal pneumolysin antibody (abcam, ab71811, Cambridge, UK). Western blotting was done according to the method used by Waltman WD et al. [28]. Briefly, the 8-h of IDM-*ply* culture were harvested after overnight pneumococci grown from 5% blood agar with erm was adjusted to OD_600_ 0.05 in 20 ml BHI broth at 37°C and 5% CO_2_ for eight hours until its OD_600_ equals to 0.35. Day 1 culture was obtained after adjusting the same OD_600_ 0.05 through inoculation the 8-h culture into another 20 ml BHI broth and was incubated at the same condition overnight. Cultures, centrifuged at 4000 rpm for 15 min, was washed twice with phosphate buffered saline (PBS), pH7.2. The pellet was then incubated with 200 μl lysis buffer with 100X Halt protease and phosphatase inhibitor Cocktail (Thermo Scientific, USA) at 37°C for 30 min. and sonicated to obtain the lysate. 40 μg protein in each lysates, measured by BCA method (Thermo scientific, USA), was run in a 10% SDS-PAGE and subsequently overlaid with a 0.2 μm PDVF membrane. Immobilon-PDVF membrane which was run by 100 mA for 1 hour and 20 min was blocked with 5% skim milk for 1 h at room temperature. Rabbit polyclonal pneumolysin antibody (1:2000) (Abcam, Cambridge, UK) was shaken overnight at 4°C. Goat polyclonal to rabbit IgG (HRP) antibody was used as secondary antibody (1:10000) was agitated for 1 hr at RT. WesternBright ECL reagent (Adansta, CA, USA) was used to detect WT PLY.

### Evaluation of *in vivo* TGR in mice

In the *in vivo* analyses, 3-h cultures of 10^2^–10^4^ CFU of IDM-*ply* and IDM-*lytA* cultivated in BHI with and without antibiotics were injected intraperitoneally into five male 6-week-old BALB/c mice (National Laboratory Animal Center, Taiwan). Mice showing signs of illness at 10 min, 1 day, and 2 days were selected and sacrificed. Blood and liver samples were collected aseptically and DNA was then extracted using a DNeasy Blood and Tissue Kit (Qiagen, Hilden, Germany). Twenty nanograms of DNA from each sample were used as template in PCR analyses to detect intact *ply* or *lytA*, indicating TGR. The respective IDM-*ply* and –*lytA* genes were also amplified as controls and used for relative quantification. All animal experiments were approved by the Institutional animal care and use committee (IACUC) of National Health Research Institutes (NHRI-IACUC-101078-A) and were carried out according to their guidelines.

### Effect of TGR on virulence in mice

To assess the effect of TGR on lethality in mice, 3-h and 8-h cultures of IDM mutants, incubated in the same liquid media as that used in the *in vivo* study, were investigated. D39 was used as the control. Ten-milliliter and 1-ml aliquots from 3-h and 8-h IDM cultures, respectively, were centrifuged and then washed with ice-cold phosphate buffered saline (PBS). Pellets were suspended in 300 μl of PBS. Ten 6–8-week-old male BALB/c mice were injected IP with approximately 10^2^ CFU of each culture and then monitored for 14 days. Differences in survival among IDM mutants were analyzed at two time points using a log-rank test in SigmaStat 3.5 software (SPSS, Chicago, IL, USA).

## Abbreviations

KD: Knockdown; KO: Knockout; ply: Pneumolysin gene; lytA: Autolysin gene; IDM: Insertion duplication mutagenesis; IFD: In-frame deletion; TGR: Target gene restoration; WT: Wild-type; qPCR: Quantitative polymerase chain reaction; DIG: Digoxigenin; cat: Chloramphenicol; erm: Erythromycin; IP: Intraperitoneal; gki: Glucose kinase gene; MHA: Muller-Hinton agar; BHI: Brain heart infusion; CFU: Colony-forming units; ml: Milliliter; T: Time point; IACUC: Institutional animal care and use committee; PBS: Phosphate buffered saline.

## Competing interests

The authors declare that they have no competing interests.

## Authors’ contributions

EYL designed the study. EYL and JCC performed the laboratory work. EYL analyzed the data. CPF supervised the study. EYL, FYC, and CPF prepared the manuscript. All authors read and approved the final version of the manuscript.
